# A Culturally Responsive Trauma-Informed Public Health Emergency Framework for Aboriginal and Torres Strait Islander Communities in Australia, Developed during COVID-19

**DOI:** 10.3390/ijerph192315626

**Published:** 2022-11-24

**Authors:** Simon Graham, Ilias Kamitsis, Michelle Kennedy, Christina Heris, Tess Bright, Shannon K. Bennetts, Kimberley A Jones, Renee Fiolet, Janine Mohamed, Caroline Atkinson, Catherine Chamberlain

**Affiliations:** 1 Department of Infectious Diseases, Peter Doherty Institute for Infection and Immunity, University of Melbourne, Melbourne, VIC 3000, Australia; 2 Indigenous Health Equity Unit, Melbourne School of Population and Global Health, The University of Melbourne, Melbourne, VIC 3000, Australia; 3 School of Medicine and Public Health, University of Newcastle, Newcastle, NSW 2308, Australia; 4 National Centre for Aboriginal and Torres Strait Islander Wellbeing Research, National Centre for Epidemiology and Population Health, Australian National University, Canberra, ACT 2601, Australia; 5 Judith Lumley Centre, School of Nursing and Midwifery, La Trobe University, Bundoora, VIC 3086, Australia; 6 Intergenerational Health Group, Murdoch Children’s Research Institute, Parkville, VIC 3000, Australia; 7 The Lowitja Institute, Collingwood, VIC 3066, Australia; 8 We Al-Li, Goolmangar, NSW 2480, Australia; 9 NGANGK YIRA: Murdoch University Research Centre for Aboriginal Health and Social Equity, Murdoch University, Perth, WA 6150, Australia

**Keywords:** Indigenous, complex trauma, Aboriginal, Torres Strait Islander, public health emergency, framework

## Abstract

The Coronavirus Disease 2019 (COVID-19) pandemic impacted peoples’ livelihoods and mental wellbeing. Aboriginal and Torres Strait Islander peoples in Australia continue to experience intergenerational trauma associated with colonization and may experience trauma-related distress in response to government responses to public health emergencies. We aimed to develop a culturally responsive trauma-informed public health emergency response framework for Aboriginal and Torres Strait Islander peoples. This Aboriginal and Torres Strait Islander-led study involved: (i) a review of trauma-informed public health emergency responses to develop a draft framework (ii) interviews with 110 Aboriginal and Torres Strait Islander parents about how COVID-19 impacted their lives, and (iii) a workshop with 36 stakeholders about pandemic experiences using framework analysis to refine a culturally responsive trauma-informed framework. The framework included: an overarching philosophy (cultural humility, safety and responsiveness); key enablers (local leadership and Eldership); supporting strategies (provision of basic needs and resources, well-functioning social systems, human rights, dignity, choice, justice and ethics, mutuality and collective responsibility, and strengthening of existing systems); interdependent core concepts (safety, transparency, and empowerment, holistic support, connectedness and collaboration, and compassion, protection and caring); and central goals (a sense of security, resilience, wellbeing, self- and collective-efficacy, hope, trust, resilience, and healing from grief and loss).

## 1. Introduction

In Australia, an estimated 10.3 million cases of Coronavirus Disease 2019 (COVID-19) and about 15,700 deaths (0.1% of COVID-19 cases) have been reported as of November 2022 [1]. Individuals aged 60 years or older, those with chronic health conditions and those who are immunocompromised have an increased risk of hospitalization or death if infected with COVID-19 [2]. Public health responses to COVID-19 aim to protect people from infection or harm, but these actions can also negatively impact people’s health and livelihoods [3]. Policies implemented in response to public health emergencies may increase fear of a disease, such as COVID-19, and therefore finding ways to protect people from infection or harm but also decrease fear and distress is an important part of a public health emergency response.

Over the past two decades, there has been an increased recognition of ‘complex trauma’ or complex post-traumatic stress disorder (CPTSD) [4,5,6,7]. CPTSD can occur in response to ‘overwhelming fear’ or a long period of exposure to severe threats where escape is not possible [6]. As our awareness and understanding of complex trauma and its negative impact has developed, a new focus on trauma-informed care and approaches has emerged, which aim to mitigate the impacts of complex trauma [8]. The COVID-19 pandemic is an example of a traumatic stressor that could increase trauma-related distress or symptoms, especially for people with a history of trauma [9,10,11,12].

In the context of public health emergencies, fear-based messaging is often used to change the behavior of populations or group and protect them from infection or harm. However, fear-based messaging has the potential to increase trauma-related distress [13]. In Australia, examples included television advertisements showing very sick people with COVID-19 in hospital and the media’s constant reporting of COVID-19 case numbers and deaths in the last 24 h [14]. The government’s objective for using these advertisements was to motivate people to stay home, wear a mask when outside their home, get tested if they have symptoms and get vaccinated; to reduce risk of disease transmission and mortality. The intention behind such messaging is that confronting images may increase personal perceived risk from exposure to COVID-19 and increase the likelihood that people will follow government advice [15]. Such communications strategies are thought to be effective only when the audience has the capacity and can implement the recommendations [15]. As such, these messages can potentially cause distress among individuals who are unable to follow the suggested behaviors (e.g., essential workers who are unable to work from home) or among sub-populations that might be at increased risk of poor health outcomes or death, e.g., older people. One study found that individuals with a history of childhood trauma were more likely to perceive COVID-19 as a greater threat, compared to people who do not have a history of childhood trauma [16]. Fear activates an instinctive attempt to escape a threat, commonly referred to as the ‘fight-or-flight’ response [8]. When the individual is unable to escape then a ‘freeze’ response can occur [17]. After the traumatic experience is over, these responses “may be reactivated at the slightest hint of anything resembling the previous threat and secrete stress hormones” [18]. Individuals caught up in this pattern of fear responses may have a limited ability to modify their behavior [19], and are likely to experience unpleasant emotions and intense physical sensations (trauma-related distress) [18]. Therefore, there is a need for trauma-informed public health approaches that can address both the public health emergency and include strategies to mitigate trauma-related distress.

Aboriginal and Torres Strait Islander peoples are the first peoples in Australia and have a rich and strong history [20]. Compared to non-Indigenous people, Aboriginal and Torres Strait Islander peoples have higher rates of chronic health conditions such as diabetes, cardiovascular disease, hypertension and cancer; which can increase their risk of poorer health outcomes from public health emergencies such as COVID-19 [9,10,11,12,21].

Aboriginal and Torres Strait Islander peoples are particularly impacted by complex trauma due to historical and intergenerational trauma associated with colonization [22,23]. This includes the impact from previous epidemics [24] and government ‘interventions’, including state-sanctioned removal of Aboriginal and Torres Strait Islander children from their families [25]. Forced separation negatively disrupted families, with many children placed in orphanages or with non-Indigenous families and in some cases were subjected to physical, sexual and emotional abuse [26]. Due to ongoing colonizing practices, Aboriginal and Torres Strait Islander communities have experienced loss of culture and land, and increased exposure to violence, discrimination, and racism [27,28,29]. A consequence of these negative historical events has been an increase in mistrust of Australian governments and government policies within Aboriginal and Torres Strait Islander communities. During the COVID-19 pandemic in Australia, this mistrust was evidenced by Aboriginal and Torres Strait Islander people having lower vaccination rates, being less likely to attend a hospital and being less likely to get tested for COVID-19 [1,30]. Similar experiences of mistrust have been reported internationally, including Indigenous communities in northern India implementing their own restrictions to stop people visiting their community and reporting reservations about being vaccinated for COVID-19 [31].

Aboriginal and Torres Strait Islander leaders and communities have increasingly advocated that Aboriginal and Torres Strait Islander peoples should be centered in the decision-making process, within a “culturally appropriate governance structure” [32]. This was a lesson learnt from the disproportionate impacts of the 2009 Swine flu pandemic in Australia [33]. In March 2020, the Australian Government’s Department of Health convened the Aboriginal and Torres Strait Islander Advisory Group on COVID-19 co-chaired by the National Aboriginal Community Controlled Health Organization to ensure that “all stages of the pandemic are considered with an equity lens, are proportional to the risk of disease in communities, to discuss and work through logistical issues related to the pandemic especially in planning phases and that these actions should be locally led, holistic and culturally safe to communities” [24].

Culturally appropriate Aboriginal and Torres Strait Islander-led strategies are critical to improving the health of Aboriginal and Torres Strait Islander peoples in Australia and ‘closing the gap’ in health inequities [24,34,35]. Aboriginal and Torres Strait Islander culture is a source of strength and resilience [34], with studies highlighting that incorporating culture into public health programs and research is integral to achieving positive outcomes [36]. Engagement with Aboriginal and Torres Strait Islander peoples “enables, reflects and integrates culture in its essence” p. 2 [35], and increases people’s trust and access to care [37]. Aboriginal and Torres Strait Islander leadership is empowering and ensures that cultural values and practices are embedded within healthcare delivery [35].

The implementation of culturally appropriate strategies, collaboration with local communities and privileging Aboriginal and Torres Strait Islander voices in public health emergencies is integral to reducing the health inequities described above. To assist in achieving this, we aimed to develop a culturally responsive trauma-informed public health emergency framework for Aboriginal and Torres Strait Islander communities in Australia.

## 2. Materials and Methods

### 2.1. Study Context

This Aboriginal and Torres Strait Islander-led research was conducted as an adjunct to an existing study called Healing the Past by Nurturing the Future (HPNF). The HPNF project aims to co-design and implement perinatal strategies for Aboriginal and Torres Strait Islander parents experiencing complex trauma [38]. We used an Indigenist participatory, mixed-methods approach which recognizes the impacts of colonization for Aboriginal and Torres Strait Islander peoples and actively aims to improve equity for Aboriginal and Torres Strait Islander peoples.

### 2.2. Study Approach

This study used participatory qualitative methods to develop the trauma-informed framework [39]. Our rationale for using this approach was that participatory methods allow Aboriginal and Torres strait Islander people freedom to discuss any issues related to our topic and also allows them to drive the design and development of the framework so that it can be used in real world contexts. To develop the framework, we used three interconnected studies: (i) a rapid review of trauma-informed public health emergency approaches to develop a draft framework, (ii) interviews with Aboriginal and Torres Strait Islander parents regarding their experiences of COVID-19, and (iii) a key stakeholder workshop to present drafted framework components and to build the final culturally responsive framework together with stakeholders.

#### 2.2.1. Rapid Review of Trauma-Informed Public Health Emergency Frameworks

Firstly, a rapid review was conducted to identify key features of previously developed trauma-informed public health emergency frameworks [40,41]. We searched relevant databases from 1 January to 30 November 2020, which yielded approximately 10,000 studies, of which 40 were included. Extracted data were subjected to framework and thematic synthesis. No studies reported evaluations of a trauma-informed public health emergency response. However, included studies highlighted key elements of a ‘trauma-informed lens’, which may help to consider implications, reduce risks, and foster a sense of security, wellbeing, self-and collective-efficacy, hope and resilience for First Nations communities during COVID-19. We identified key elements for minimizing the impact of compounding trauma on Aboriginal and Torres Strait Islander communities, including: a commitment to equity and human rights, cultural responsiveness, good communication, and positive leadership. The principles guiding trauma-informed culturally responsive public health emergency frameworks included: safety, empowerment, holistic support, connectedness and collaboration, compassion and caring, trust and transparency in multi-level responses, well-functioning social systems, and provision of basic services. Detailed methods are outlined elsewhere (the review has been accepted for publication) [40].

#### 2.2.2. Interviews with Aboriginal and Torres Strait Islander Parents

Secondly, as part of the HPNF project [38], a survey and interviews were conducted between 1 August 2020 and 30 May 2022 with 110 Aboriginal and Torres Strait Islander parents from Victoria, South Australia (SA), and the Northern Territory (NT), Australia. The survey collected quantitative information such as demographics, trauma symptoms, experiences of COVID-19, the impact of COVID-19 on their livelihoods, their social and emotional wellbeing, their relationships, and their community connection. During the interviews, parents were asked about their experiences of COVID-19 using open-ended questions to collect additional qualitative information. Detailed methods are provided elsewhere (manuscript is under review).

#### 2.2.3. A Workshop to Discuss Findings with Key Stakeholders

Thirdly, we conducted a two-day online workshop in October 2021. The aim of this workshop was to co-design key components for inclusion in a culturally responsive trauma-informed public health emergency framework for Aboriginal and Torres Strait Islander peoples. The workshop included discussions of our rapid review and draft framework, results from the interviews with Aboriginal and Torres Strait Islander parents and presentations from other researchers working with Aboriginal and Torres Strait Islander communities during the COVID-19 pandemic. Invitations to participate in the workshop were sent through professional networks, including the Australian Partnership for Preparedness Research on Infectious Disease Emergencies (APPRISE) and the HPNF project, the Lowitja Institute (an Aboriginal and Torres Strait Islander health and wellbeing organization with extensive national networks) mailing lists and social media. Participants were asked to complete a registration form on REDCap [42], read a Participant Information Statement and sign a consent form. Due to government COVID-19 restrictions, the workshop was held via Zoom. Thirty-six health professionals, academics and community leaders from 18 institutions across Australia attended day one of the workshop, with 83% returning for day two. Over 50% of participants were Aboriginal and Torres Strait Islander people.

#### 2.2.4. Workshop Planning to Create a Safe Environment

**Figure 1:**
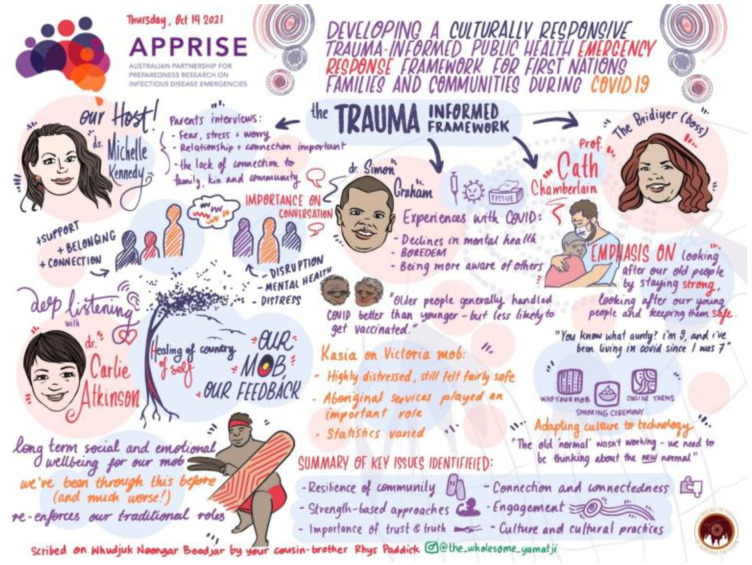
Graphic design of the trauma-informed workshop.

Strategies to ensure cultural and emotional safety are critical in any trauma-informed work [43]. Prior to the workshop, participants were sent a pack which included: a trauma response factsheet and information for psychological support if required, diversionary activities, such as mindfulness coloring, modelling beeswax, a water bottle and refreshments. At the start of the workshop, a trained facilitator guided the group through a ‘Dadirri’ (deep listening) exercise and encouraged participants to use this throughout the workshop to help with self-regulation. Four guiding principles were introduced to foster a safe space for engagement (i.e., confidentiality, being respectful, being brave, and being kind), and activities such as stretching, music and yoga were used throughout the workshop to assist participants to regulate any feelings of trauma-related distress. Participants were introduced to a psychologist at the start of the workshop who was available for consultation by phone if needed. An Aboriginal graphic facilitator was engaged to integrate respectful humor and capture key elements of the discussion visually (Figure 1). See Supplementary Material Figure S1 and Supplementary Material Figure S2 for an outline of the workshop.

#### 2.2.5. Workshop Procedure

Prior to the workshop, participants were sent a copy of the rapid review and a related plain language article published in the media [41]. The workshop was facilitated by an Aboriginal (Wiradjuri) woman who is also an investigator on the project (MK). On day one, six presenters shared research findings about Aboriginal and Torres Strait Islander communities’ experiences of the COVID-19 pandemic. The studies were conducted across a variety of populations, including remote Aboriginal and Torres Strait Islander communities, Stolen Generations survivors, Aboriginal and Torres Strait Islander people living in Western Sydney and Victoria, and Aboriginal and Torres Strait Islander parents from the Victoria, South Australia and the Northern Territory [44]. Participants were allocated to Zoom breakout rooms, where they discussed the content of the presentations and reflected on what components should be included in a public health emergency response framework. Their ideas about key features of the framework were shared on the online platform “Poll Everywhere” and reflected in real time using a word cloud. Participants voted on the importance of framework components, which were then ranked in order of importance. Connections between components and relevant considerations about the framework were discussed in a whole group forum.

On day two of the workshop, CH presented the results of a rapid review of studies identifying key principles and features of trauma-informed public health emergency responses. Participants discussed these findings in breakout rooms and considered whether any components identified in the review should be added to our framework. Framework components identified in the review were collated with the components identified by participants on day one and presented for further discussion in a whole group forum. Participants also considered the visual presentation of the framework and how components relate to each other. The workshop was video-recorded and detailed notes were taken by members of the research team during breakout room discussions on both days. Following the workshop, all attendees were invited to contact the research team if there were other contributions they wished to make. A draft workshop report was sent to all participants, asking for feedback as to whether the report reflected views shared. All workshop participants were invited to be co-authors on this report or included in an acknowledgement.

#### 2.2.6. Workshop Data Analysis

**Figure 2:**
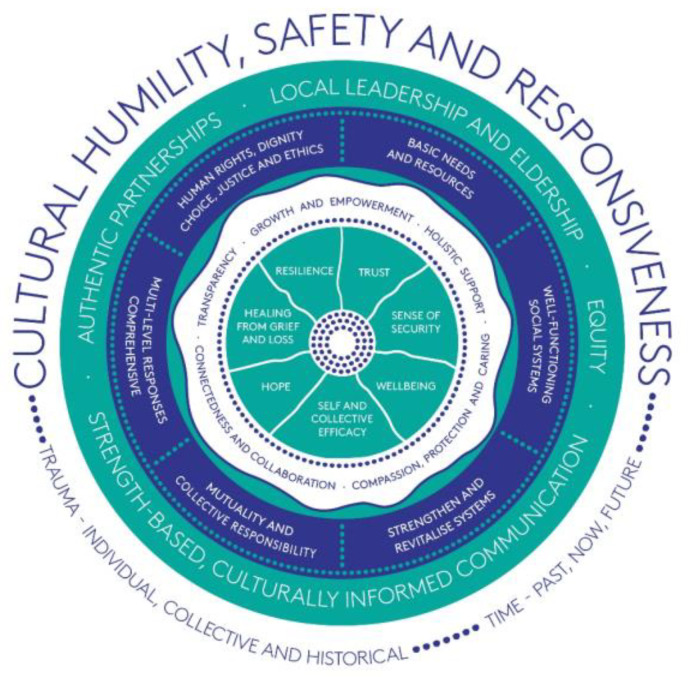
A culturally responsive trauma-informed public health emergency framework for Aboriginal and Torres Strait Islander communities.

Workshop discussions in the whole group forum were transcribed. Breakout room discussion notes and transcripts were entered into the qualitative data analysis software program NVivo [45], and coded using ‘best fit’ framework analysis [46]. This approach is relatively rapid, transparent, and pragmatic, whereby predetermined codes were set in NVivo derived from the previous rapid review and workshop discussions, with an ‘other’ code. Workshop data were then coded against these predetermined codes where they ‘best fit’, and data that did not fit a predetermined code was coded to ‘other’, and subsequently coded using inductive thematic analysis with members of the authorship team to refine the final framework (Figure 2). The research team met twice to discuss the coding and framework analysis to ensure the team was capturing all the messages and themes.

### 2.3. Ethics

This study received ethics approval from the following committees in three States and territories of Australia: Victoria: The St Vincent’s Hospital (HREC 060/20); Department of Health and Human Services, La Trobe University; Royal Women’s Hospital; University of Melbourne); in South Australia: The South Australian Aboriginal Ethics Committee (AHREC 04-20-873) and The Women’s and Children’s Health Network and in the Northern Territory: The Central Australian Human Research Ethics Committee (CA-20-3705).

## 3. Results

An overview of the trauma-informed public health emergency framework for Aboriginal and Torres Strait Islander communities is illustrated in Figure 2. It has elements of time and trauma weaving through five layers:

1. Central goals
2. Interdependent core concepts
3. Supporting strategies
4. Key enablers
5. An overarching philosophy

We describe core themes and related concepts below:

### 3.1. Level 1: Central Goals

Seven central goals of a trauma-informed emergency response include facilitating: (1) a sense of security; (2) healing from grief and loss; (3) hope; (4) resilience; (5) self and collective efficacy; (6) trust; and (7) wellbeing.

Workshop participants emphasized the importance of having a sense of security or safety, which can be achieved through structured practical support, creating community spaces where people feel safe, and implementing a trauma-informed approach when engaging with families and communities. Participants noted that during the COVID-19 pandemic, restrictions and lockdowns interfered with people’s ability to hold a grieving space which led to unresolved collective grief. It was reported that in Victoria and New South Wales (NSW), many communities experienced COVID-19 immediately after the 2019 bushfires, compounding the sense of trauma, grief and loss. Healing from grief and loss was thus identified as an essential outcome of applying the framework.

*In NSW, many communities went straight from bushfires to COVID; there just wasn’t that time to grieve. Sorry business has been continuous, community still have not overcome the effect of the bushfires.*

Participants proposed that community strengths have helped people survive and build resilience during public health emergencies. Participants reported that in the research studies presented during the workshop, there were messages of survival, strength and endurance, and these traits were supported by a connection to Country, culture and community. However, it was also noted that some families did struggle during the pandemic, and that finding ways to enhance resilience in those individuals was important. Self and collective efficacy was also identified as an important component that can help build resilience.

*[It’s] important for young people to be involved in the response and have some control and responsibility. This will also build their resilience.*

Trust was identified as a key component to building strong relationships with communities. However, participants reported that due to trauma associated with government policies and actions, for some community members, mistrust in government existed. Redressing historical and contemporary trauma was thus considered necessary if there is to be any trust in a relationship with community.

*I think the relationship and the engagement part go really strongly with trust, so if you think about what forms a really strong relationship, trust is usually a key component to that.*

Participants spoke about the importance of recognizing that different groups of people have been affected in different ways during the pandemic. Many women experienced difficulties relating to birthing due to limited support, including restrictions on family members being able to attend births. Children and young people experienced significant developmental milestones while living in an unpredictable world that is changing rapidly, such as finishing school, starting university or becoming parents for the first time. It is thus important to consider ways of maintaining the wellbeing of different groups of people during public health emergencies.

*[We] need to think about the impacts on adolescents and the impact on their self-esteem. Also, the impacts on the under 5 age group, including parental anxiety and loss of social connections. There needs to be recognition that different age groups are affected in different ways.*

### 3.2. Level 2: Interdependent Core Concepts

The framework includes the following five interdependent core concepts that support measures to achieve the central goals: (1) compassion, protection and caring; (2) connectedness and collaboration; (3) growth and empowerment; (4) holistic support; and (5) transparency.

The importance of maintaining compassion, protection and caring for individuals and families was emphasized. Participants shared instances where an inability to perform cultural practices and a lack of compassion from hospital staff during the pandemic resulted in increased distress for them. One participant spoke about an incident at a hospital:

*The situation would have been less distressing if the hospital staff took the time to listen and understand and respect the family’s needs and wishes. If there was more compassion in their response.*

Participants discussed the tragic loss of life and grief associated with the pandemic. Therefore, consideration needs to be given to palliative care and the long-standing traditions and cultural rituals practiced at end of life, such as returning to Country. Participants further reported that having a sense of connectedness to culture, community and family is fundamentally important to Aboriginal and Torres Strait Islander peoples’ wellbeing. However, maintaining this connection was challenging during the pandemic, as lockdowns and restrictions resulted in cultural events being cancelled and people being unable to see family members in person. While some people found alternative ways to stay connected to community and family (e.g., Zoom), others struggled to connect. The importance of collaboration and coming together as a community was also noted. This can be achieved through the use of inclusive rather than divisive language when discussing important topics.

*I think one of the biggest challenges that we do have at the moment is with people being a bit hesitant about vaccines and having understandable lack of trust, is how do we keep them connected and not feel othered, …and the importance of having inclusive and not divisive language and ways of talking and way of being in this pandemic and helping everybody come through it together.*

Participants mentioned that positive health messaging (i.e., focusing on what people are able to do, rather than focusing on restrictions) can enhance community self-determinism, growth and empowerment. Emergency responses that strengthen and empower communities can aid recovery from the pandemic. Participants reported that transparent communication between government agencies and communities is essential. They also spoke about the need for holistic support for communities. This could include interventions that help de-escalate trauma-related symptoms, promotion of the benefits of exercise and pets, physical therapies and plant-based medicine.

*What was missing to some extent was discussion around the necessity for positive health messaging, particularly when a lot of the messaging since [the] outbreak has been focused on what people cannot do, which has an impact on self-determination and agency.*

### 3.3. Level 3: Supporting Strategies

The framework includes six practical supporting strategies: the provision of (1) basic needs and resources; (2) human rights, dignity, choice, justice and ethics; (3) multi-level comprehensive responses; (4) mutuality and collective responsibility; (5) strengthen and revitalize existing systems; and ensuring (6) well-functioning social systems.

Participants reported that during the pandemic it is necessary for communities to have their basic needs and resources met, such as access to consistent water supply and sanitation facilities. People also need to be informed about vaccination in simplified and easily accessible formats. Participants further spoke about the importance of helping people to maintain their dignity and be offered choice and ensuring that human rights, justice and ethics are respected.

*We need more on choice/the right for choice—important aspect of cultural integrity. Perhaps belongs between communication and equity and human rights.*

Participants highlighted the need for multi-level responses, as well as the importance of government/health agencies and community organizations working together during the pandemic. Participants emphasized the importance of strengthening and revitalizing existing cultural and social systems, and building mutuality and collective responsibility by giving communities the opportunity to grow and build on their strengths. Participants felt this could create a sense of achievement and allow for systemic change for Aboriginal and Torres Strait Islander people in Australia. However, participants also emphasized the need for targeted funding for culturally appropriate, community-led prevention programs.

### 3.4. Level 4: Key Enablers

The framework includes four key enablers that underpin supporting strategies: (1) maintaining authentic partnerships and investing in ongoing relationships; (2) equity; (3) local leadership and Eldership; and (4) strengths-based culturally informed communication.

Participants highlighted the importance of maintaining authentic partnerships with community-controlled health organizations during the pandemic. Concerns were also expressed about inequitable treatment in the COVID-19 response. For example, professional footballers were able to cross state borders while families and communities where unable to undertake important cultural practices, including attending funerals. This experience can be reminiscent of past inequities, and equity needs to be considered when responding to public health emergencies.

Participants suggested that leadership and Eldership needs to come from community and highlighted the importance of engaging Elders and acknowledging their cultural knowledge and life experience. They further mentioned that within communities there are existing leaders, such as Elders, and emerging young leaders, who can provide critical support during the pandemic.

*It is important to capture the role of Elders in supporting the younger generation and also the role that the younger generation has in supporting Elders.*

Participants reported that there was a need to focus on reframing the narrative during the pandemic. Strength-based culturally informed communication which focuses on how people can stay healthy and in control of their lives would be more self-determining than a punitive approach outlining repercussions for breaches of restrictions. Participants highlighted the importance of determining how to best facilitate safe and constructive ways of engaging with community members who are angry or ambivalent, and conveying information in ways that reduce fear. Identifying high quality and reliable Aboriginal and Torres Strait Islander information sources was also viewed as important, especially for topics such as the effectiveness of vaccines.

### 3.5. Level 5: Overarching Philosophy

The group of predominantly Aboriginal and Torres Strait Islander participants asserted that an overarching philosophy of cultural humility, safety and responsiveness should inform everything within the framework. Participants specifically reported that cultural practices and rituals are integral to enhancing the social and emotional wellbeing of Aboriginal and Torres Strait Islander people, and there needs to be strategies to maintain these as much as possible during public health emergencies and responses.

### 3.6. Elements which Weave through Each Layer

#### 3.6.1. Time—Past, Present and Future

Participants described the importance of considering public health emergency responses through a lens which recognizes the importance of time, including historical events impacting communities from the past, the present context, and the need to act in the best interest of future generations to come.

#### 3.6.2. Trauma—Individual, Collective and Historical

During the workshop, Aboriginal and Torres Strait Islander participants discussed the need to recognize many different types of trauma, including individual (complex trauma), collective and historical trauma, and this needs to be considered throughout the concepts outlined in the framework. It was noted that government restrictions during the pandemic could for some people trigger memories of traumatic experiences resulting from past government actions, and that past trauma may exacerbate the negative psychological impact of COVID-19. Moreover, participants mentioned that by helping people understand trauma and its impact on the wellbeing of Aboriginal and Torres Strait Islander people, the framework can potentially allow organizations to become trauma-integrated whereby they consistently apply trauma-informed principles and support in their service provision going forward.

## 4. Discussion

We present a culturally responsive trauma-informed public health emergency framework for Aboriginal and Torres Strait Islander communities, co-designed by and with Aboriginal and Torres Strait Islander people, informed by three data collection methods. The framework provides a ‘lens’ through which to view public health emergency responses and consider ways to minimize trauma-related distress and increase community cooperation with a focus on how, not what, to do.

Aboriginal and Torres Strait Islander leadership has been critical to the success of the COVID-19 response and mitigation of negative impacts on Aboriginal and Torres Strait Islander communities in Australia [32]. Using a trauma-informed lens, this framework describes the multi-layered and critical components required to implement a culturally responsive public health emergency response. It provides important learnings from Aboriginal and Torres Strait Islander leaders and community members, for future public health emergencies. Trusted sources for Aboriginal and Torres Strait Islander peoples, such as community-led services (e.g., Aboriginal Community Controlled Health Services (ACCHS)) were key to successful messaging during the pandemic, including where and how to be tested, where and why community members should be vaccinated, and explaining COVID-19 treatments [1]. This highlights that for future pandemics, and for other health messaging more generally, governments and general practitioners need to collaborate with Aboriginal Health Services, leaders, or community organizations to have impact or success.

The framework also included the role of Elders and the need to protect them as most vulnerable to risks of COVID-19. Elders played a key role in the COVID-19 health messaging and promotion campaigns, such as mask wearing, being tested, where to go for testing and promoting vaccination [32]. Having Elders advocate for COVID-19 vaccination was a powerful tool to motivate Aboriginal and Torres Strait Islander peoples. In October 2022, the Australian Department of Health reported that 81% of Aboriginal and Torres Strait Islander peoples were vaccinated for COVID-19, the tipping point in reaching herd immunity [47]. A study of 35 Aboriginal and Torres Strait Islander people living in Western Sydney highlighted that one motivation for young Aboriginal and Torres Strait Islander peoples to get vaccinated and wear a mask was the protection from COVID-19 infection these actions offered their communities, especially their Elders [1]. This study also found that Aboriginal and Torres Strait Islander people had very high levels of trust in their local ACCHS and they preferred to access COVID-19 information, testing and vaccination through the ACCHS. Especifically for Aboriginal and Torres Strait Islander peoples, feeling safe and and having cultural safety in health care is vital to improving health outcomes [30]. This is in line with studies showing the importance of feelings of safety and trust regarding vaccination and health measures during COVID-19 [48].

Authentic partnerships have been highlighted to improve not only working relationships but health outcomes for Aboriginal and Torres Strait Islander communities in Australia [49,50]. One example is the Miwatji Leadership Model, which respects local Yolngu forms of authority and empowers the community to develop, manage and sustain their own health. It also supports formal ways to take on leadership roles, which has increased both accessibility and acceptability of health care services [50]. Another study which conducted 20 interviews with Aboriginal and Torres Strait Islander people living in a remote area of the Northern Territory highlighted that the community highly valued deep reciprocal relationships with Country and ecological knowledge, strong kinship relations and Aboriginal and Torres Strait Islander-led community organizations, as these enabled disaster risk reduction [51]. The workshop highlighted that authentic and ongoing reciprocal relationships and partnerships were desired, and these relationships also improved the chances of success and benefits for communities.

Our framework suggests that the following concepts need to be central goals during responses compassion, protection and caring, connectedness and collaboration, growth and empowerment, holistic support and transparency. These features were also important in a review examining physical injuries during a disaster or emergency, which were easier to identify and address compared to mental health distress from a disaster or emergency [52,53,54]. It found that about 40% of individuals that attended shelters and family assistance centers due to a disaster had pre-existing mental disorders and these conditions were exacerbated due to a public health emergency [52,53,54]. Another study which included interviews with leaders in disaster management in Canada, Australia, the United Kingdom, and the United States found six overarching themes were key to success: merging traditional and modern approaches, community engagement, connectedness and relationships, investing in preparedness, putting knowledge into practice and ensuring human and financial resources [53]. This suggests that our framework could provide real life benefits with connectedness and holistic approaches to public health emergencies.

Other studies also found that these concepts helped to reduce the impact of public health emergencies on trauma [55,56,57]. One example is the process used by the San Francisco Department of Health to develop and implement their Trauma-Informed Systems Initiative at an organizational level to address trauma at the systems level [56]. Their initiative includes principles such as safety and stability, understanding trauma and stress, resiliency, and recovery. Healing from grief, resiliency and having trust facilitates a process that allows people’s voices to be heard and increases confidence to be able to reduce trauma from a public emergency event.

A major strength of our study was that it had three data collection methods, all co-designed with and led by Aboriginal and Torres Strait Islander peoples. We also followed a strength-based approach in privileging Aboriginal and Torres strait Islander voices and experiences. Our workshop was attended by Aboriginal and Torres Strait Islander people from each state and territory in Australia to gain a range of views and experiences. As our project was about trauma-informed approaches, we also used a trauma-informed approach to our interviews with parents and when conducting the workshop to ensure safety.

### 4.1. Limitations

There are some limitations to consider. The workshop was conducted online during the pandemic, which may have limited active participation of all workshop attendees. Due to the timing of the workshop, the discussion and experiences was heavily influenced by the COVID-19 pandemic, with less emphasis on other types of public health emergencies such as floods and bushfires. Our framework has not yet been validated in a public health emergency setting, and it is likely that it will need to be adapted for different emergencies and contexts. However, a co-author (CA) has reflected on the framework during a subsequent response to devastating floods in a local community and the core elements appear to be highly relevant for this context. This is a comprehensive framework and it is possible not all elements require consideration in all emergencies in all settings. However, this framework offers a ‘lens’ to consider whether a response is culturally responsive and trauma-informed and potentially identify areas for improving the effectiveness of the response. Components in our framework will need to be tested and evaluated during a public health emergency to assess their feasibility of implementation.

### 4.2. Implications for Practice

This framework provides a lens for considering culturally responsive trauma-informed approaches to public health emergencies within Australia, including floods and bushfires associated with climate change. One of the study authors (CA) has reflected on the framework in establishing a healing center in northern NSW (the Northern Rivers Community Healing Hub) to provide support for local community members during and after major floods in that region [58]. This has and continues to severely impact people’s health and livelihoods including high rates of anxiety, depression and distress. CA implemented Aboriginal and Torres Strait Islander ways of yarning, storytelling, creative activities to build connections and mutual support to reduce levels of distress [59]. This example demonstrates the use of our trauma-informed framework during other public health emergencies.

### 4.3. Implications for Policy

Our framework has implications for policy, not only for how to respond to public health emergencies with Aboriginal and Torres Strait Islander communities but for preparedness. The framework can help build appropriate policies, approaches and guidelines to mobilize and respond quickly to Aboriginal and Torres Strait Islander communities with wellbeing at its center. Preparedness is all about being ready and our framework can assist in the real world to reduce the risks of trauma from a public health emergency. Some of Australia’s current preparedness policies are from lessons learnt from the 2009 Swine flu outbreak. Similarly, in 2022 the Australian government commissioned a review of its response to the COVID-19 pandemic which will provide recommendations and the opportunity to learn from what worked and what did not. Initial findings from the COVID-19 review highlighted inequalities in Australia’s response, especially with vulnerable populations [60].

Although our study focused on Aboriginal and Torres Strait Islander people and was based in Australia, our framework could offer a ‘starting point’ for other Indigenous populations internationally seeking to co-design their own trauma-informed public health emergency framework. It is also possible that rich Indigenous insights such as lived experience drawing on cultural knowledge are relevant for other populations globally affected by trauma and public health emergencies. One example of using culture to mitigate anxiety, depression and suicidal thoughts through interventions that place Indigenous culture at its core comes from First Nations communities in Canada [61].

## 5. Conclusions

The framework presented in this paper has been developed by an Aboriginal and Torres Strait Islander-led team and has been informed by co-design processes. The culturally responsive trauma-informed public health emergency framework for Aboriginal and Torres Strait Islander communities provides a lens for those implementing public health emergency responses (i.e., the ‘what’) to consider important elements and strategies to minimize trauma-related distress and maximize community engagement to address the ‘how’ to approach public health emergencies and not just describe them.

## Data Availability

Data used in this study is available if the Aboriginal Research Committee approves its release.

## Supplementary Materials

The following supporting information can be downloaded at: https://www.mdpi.com/article/10.3390/ijerph192315626/s1, Supplementary Material Figure S1: Workshop outline-day 1; Supplementary Material Figure S2: Workshop outline-day 2.
