# Supplementary material for: A Culturally Responsive Trauma-Informed Public Health Emergency Framework for Aboriginal and Torres Strait Islander Communities in Australia, Developed during COVID-19
Source: Int J Environ Res Public Health. 2022 Nov 24;19(23):15626. doi: 10.3390/ijerph192315626 (PMC9738974; doi:10.3390/ijerph192315626)
Supplement: Supplementary file 1 [file ijerph-19-15626-s001.zip › ijerph-2042305-supplementary.pdf]

**Supplementary material figure S1:** Workshop outline-day 1

APPRISE
AUSTRALIAN PARTNERSHIP FOR PREPAREDNESS RESEARCH ON INFECTIOUS DISEASE EMERGENCIES

WORKSHOP PROGRAM

HEALING THE PAST BY NURTURING THE FUTURE

**Thursday 14th & Friday 15th October 2021**

***Developing a culturally responsive trauma-informed public health emergency response framework for First Nations families and communities during COVID-19***

## Day 1: Thursday 14th October 2021

**Informal coffee and chat from 9.45am – Set up zoom background of favourite or Country (and add mob/Country you are on in zoom name)**
and 'Aboriginal relaxation' music https://open.spotify.com/playlist/4ajE0iinVDeT1a519nBBSU#login

| | | |
|---|---|---|
| 10:00 | **START**<br>• Acknowledgement to Country<br>• Cultural grounding exercise (Dr Caroline Atkinson) https://www.youtube.com/watch?v=tow2tR_ezL8 (Dadirri 3 mins) | Dr Michelle Kennedy (facilitator)<br>Sharing what doing that is fun or gives joy during pandemic/ share screensaver content |
| 10.20<br>(20 mins) | **Introduction, aims of the project, and purpose of the day**<br>▪ Why a First Nations trauma-informed public health emergency response framework?<br>Discussion - 10 mins | Dr Carlie Atkinson |
| 10.40<br>(10 mins) | **Creating our safe space and getting to know each other**<br>▪ Ice breaker<br>Introductions to the group and reflect on why they are here | Prof Cath Chamberlain |
| 10.50 (10 mins each) | ▪ Community experiences of COVID-19 (Dr Mark Wenitong, Lowitja Institute)<br>▪ Stolen generation experiences (Ms Jo Thitchener, Healing Foundation) | |
| 11.10-11.30 | **MORNING TEA**<br>**Stretching video:** "2 min stretching routine at the office" PhilaMassages 2013: https://www.youtube.com/watch?v=TCiEB_ahzc8 | |
| 11.30<br>(10 mins each) | **Sharing and understanding First Nations communities' experiences of COVID-19**<br>▪ Perspectives of adolescents in Western Sydney (Dr Simon Graham)<br>▪ Experiences of COVID-19 in Victoria (Ms Kasia Wojcik and Dr Jane Goller)<br>▪ Parents' experiences (Dr Michelle Kennedy)<br>▪ Summary of parent experience data (Dr Christina Heris and Dr Shannon Bennetts) | Summary written up by graphic facilitator to share |
| 12.10<br>(30 mins) | **Break out room group discussions 1: experiences**<br>▪ Experiences of group – reflect on what they have heard and key issues that **may not** have been covered. | Breakdown name for each team<br>Notes to share |
| 12.40<br>(1 hour) | **LUNCH**<br>And https://www.youtube.com/watch?v=tAUf7aajBWE (6 mins, Yoga at your desk with Adrienne)<br>(Update pics with Rhys) | |
| 1.40pm<br>(30 mins) | **1: framework components** (continued)<br>▪ Summary of key issues identified<br>▪ What are the key features of a culturally-responsive trauma-informed public health emergency framework? Goals, enablers etc. | Break out rooms 5-6 people |
| 2.10 | **Sharing and collating frameworks components**<br>▪ And ranking (poll everywhere/cahoots) | Collate into diagram |
| 2.50 | **Reflections on the day (including with Rhys)** | |
| 3.00 | **THANK YOU AND CLOSE**<br>Mini Meditation: desk break - Classic Flow - ABC Radio (4 mins) | |

**Supplementary material figure S2:** Workshop outline-day 2

## WORKSHOP PROGRAM

### Day 2: Friday 15th October 2021

**Registration and coffee from 8.45am (and sharing Rhys' summary picture)**

**Music:** RN's Nature Track https://www.abc.net.au/radionational/programs/nature-track/gippsland-rainforest-lyrebird-morning/13481746

| | | | |
|---|---|---|---|
| **9.00** | START | **Acknowledgement to country** | Dr Michelle Kennedy |
| **9.05** | **Visual recap of previous day (Rhys) and plan for today (Cath)** | | Rhys/Cath Chamberlain |
| **9.10** | **Findings from a systematic review of trauma-informed public health emergency frameworks**<br>▪ Presentation of Systematic Review findings<br>▪ Overview of HPNF conceptual framework and domains | | Dr Christina Heris |
| **9.40** | Discussion about domains from systematic review cover the issues identified yesterday and whether we need to add additional domains | | Break out rooms |
| **10.00** | Share findings of break out rooms and new things to add to conceptual framework ideas | | |
| **10.30** | MORNING TEA<br>https://www.abc.net.au/radio/programs/classicflow/mini-meditation-muscle-relaxation/10701684 4min | | |
| **10.50** | Overview of framework domains and shaping into conceptual framework<br>**Visual recap by Rhys and Cath** | | |
| **11.40** | Next steps – cultural grounding exercise and self-care tips (including Karabena self-care course https://courses.karabenacoaching.com ) | | Carlie and Michelle |
| **11.50** | THANK YOU, EVALUATION link, AND CLOSE https://redcap.link/lv808k9b | | |

**12.00-12.45 pm Optional Wayapa Yoga with Lee Couch**

**Thank you for attending the APPRISE Workshop.**

We hope you've had the opportunity to share and learn in this co-design process. It is important to look after yourself when working in trauma, so we encourage you to spend some time after this workshop in ways that are meaningful to you, for your own self-care. This might include spending time; with family, sitting and playing with children, or watching children playing; in nature, breathing in, taking off your shoes and physically grounding yourself in the dirt or sand; preparing healthy food and drinking lots of water; as well as all the different ways we move our bodies through caring, work, housework, and exercise.

**Support available at APPRISE Workshop**
